# Employer-Sponsored Health Insurance Premium Cost Growth and Its Association With Earnings Inequality Among US Families

**DOI:** 10.1001/jamanetworkopen.2023.51644

**Published:** 2024-01-16

**Authors:** Kurt Hager, Ezekiel Emanuel, Dariush Mozaffarian

**Affiliations:** 1 Department of Population and Quantitative Health Sciences, UMass Chan Medical School, Worcester, Massachusetts; 2 Food Is Medicine Institute, Friedman School of Nutrition Science and Policy, Tufts University, Boston, Massachusetts; 3 Perelman School of Medicine, University of Pennsylvania, Philadelphia; 4 The Wharton School, University of Pennsylvania, Philadelphia; 5 Division of Cardiology, Tufts Medical Center and Tufts University School of Medicine, Boston, Massachusetts

## Abstract

**Importance:**

Costs of employer-sponsored health care benefits have increased faster than workers’ wages for several decades, with important implications for disparities in earnings and wage stagnation.

**Objective:**

To quantify how growth in employer-sponsored health insurance (ESI) premiums may have been associated with reduced annual wages, disparities in earnings by race and ethnicity and wage level, and wage stagnation among US families with ESI.

**Design, Setting, and Participants:**

In this economic evaluation, serial cross-sectional analyses were performed of US families receiving ESI from 1988 to 2019 based on data from the Consumer Expenditure Survey, Kaiser Employer Health Benefits Survey, US Census Bureau’s Current Population Survey, and federal payroll taxation rates. Statistical analysis was conducted from February 2022 to July 2023.

**Main Outcomes and Measures:**

Percentage of annual compensation associated with health care premiums (after accounting for tax deductibility) and lost wages associated with growth in cost of premiums from 1989 to 2019 based on 1988 compensation. To assess disparities, analyses were stratified by race and ethnicity and wage level.

**Results:**

In 1988, 44.7 million individuals (head of household: mean [SD] age, 43.3 [13.1] years; 30.1% were female; and 2.4% identified as Asian, 6.2% as Hispanic, 8.6% as non-Hispanic Black, and 82.8% as non-Hispanic White) were covered by ESI family plans; this number remained similar in 2019 at 44.8 million individuals (head of household: mean [SD] age, 47.1 [12.9] years; 41.3% were female; and 1.3% identified as Asian, 9.9% as Hispanic, 9.9% as non-Hispanic Black, and 78.9% as non-Hispanic White). In 1988, the mean (SD) household size was 3.3 (1.3) people, and in 2019, it was 3.4 (1.3) people. If ESI costs had remained at the same proportion of the 1988 average compensation package, then in 2019, the median US family with ESI could have earned $8774 (95% CI, $8354-$9195) more in annual wages. During all 32 years, health care premiums as a percentage of compensation were greater for non-Hispanic Black and Hispanic families than for non-Hispanic White families. By 2019, 13.8% (95% CI, 13.5%-14.1%) of compensation among non-Hispanic White families with ESI went to premium costs compared with 19.2% (95% CI, 18.8%-19.7%) among non-Hispanic Black families and 19.8% (19.3%-20.3%) among Hispanic families with ESI. In 2019, health care premiums as a percentage of compensation at the 95th percentile of earnings for families with ESI were 3.9% (95% CI, 3.8%-4.0%) compared with 28.5% (95% CI, 27.8%-29.2%) at the 20th percentile of earnings. From 1988 to 2019, the mean cumulative lost earnings associated with growth in health care premiums for the median US family with ESI was $125 340 (95% CI, $120 155-$130 525) in 2019 dollars, 4.7% of earnings over the 32-year period.

**Conclusions and Relevance:**

This economic evaluation of US families receiving ESI suggests that 3 decades of increasing health care premiums were likely associated with reduced annual earnings and increased earnings inequality by race and ethnicity and wage level and were meaningfully associated with wage stagnation.

## Key Points

**Question:**

What is the association of increasing health care premium costs with earnings inequality and wage stagnation among US families with employer-sponsored health insurance?

**Findings:**

In this economic evaluation of US families receiving employer-sponsored health insurance, the mean cumulative lost earnings from 1988 to 2019 associated with growth in health insurance premiums was $125 340 per family (in 2019 dollars) or nearly 5% of total earnings over the 32-year period. In all 32 years of the study, health care premiums as a percentage of compensation were significantly higher for non-Hispanic Black and Hispanic families than for non-Hispanic White families.

**Meaning:**

This study suggests that increasing health insurance premium costs are likely associated with decreased earnings and increased income inequality, including by race and ethnicity, among US families receiving employer-sponsored health insurance and are meaningfully associated with wage stagnation.

## Teaser

This economic evaluation study quantifies how growth in employer-sponsored health insurance premiums may have been associated with reduced annual wages, disparities in earnings by race and ethnicity and wage level, and wage stagnation among US families.

## Introduction

Health disparities and affordability of health care are 2 major challenges affecting US society. Half of US adults believe health care costs are difficult to afford, with differences in affordability reported by race and ethnicity.^1^ More than half of individuals in the US receive health insurance through an employer,^2^ and the costs of employer-sponsored health care insurance (ESI) have increased faster than the overall economy for decades.^3^ The COVID-19 pandemic has reemphasized profound health inequities in the US, forcing the medical community to reevaluate how inequities are maintained today.

A potential important yet understudied association between health care affordability and health disparities in the US is indivduals’ stagnant or even decreasing incomes. Since the 1980s, real wages have increased among the highest earners but have been flat for most workers,^3^ leading to a widening earnings inequality.^4^ During the same period, the costs of employer-paid health care benefits have also increased substantially.^5^ As health economists demonstrate, it is generally accepted that increasing health care premiums result in lower wages for employees.^6,7,8,9,10,11^ Furthermore, most employers do not adjust the health care premiums charged to workers by employee earnings^12^; thus, the displacement of wages owing to increasing health care premiums could be particularly problematic for lower-wage workers^13^ and could be associated with earnings inequality.^14,15^ Little is known about the quantifiable associations with earnings inequality, including by race and ethnicity, of increasing health care premiums among US families with ESI.

We investigated 3 underlying questions about the increase in health insurance premiums. First, over 32 years beginning in 1988, how much of annual employee compensation do ESI premiums consume? Second, to what extent has the increase in ESI premiums exacerbated earnings inequality among workers receiving ESI, including by race and ethnicity and wage level? Third, over the 3 decades prior to the COVID-19 pandemic, how much have workers lost in wages due to increases in ESI premium costs? To address these questions, we combined several national data sources to analyze trends among workers with ESI family plans in employer-sponsored health care premiums, wages, and taxation, for 32 years from 1988 to 2019, overall and stratified by earnings bracket and race and ethnicity.

## Methods

### Study Design and Participants

In this economic evaluation, we used a serial cross-sectional analysis of national data to combine information on US family earnings (wages) among those with ESI, ESI premium costs, and payroll taxation from 1988 to 2019 for our overall sample and stratified by race and ethnicity and wage level. We assessed this period because 1988 was the first year in which the US Census began including Asian as a racial category, and 2019 was the last full year of data prior to large disruptions in employment, wages, and health care coverage caused by the COVID-19 pandemic. We included data on wages only among US families with ESI and did not include families with other health insurance, such as Medicare or Medicaid. All dollar amounts were adjusted for inflation and are reported in 2019 dollars. This investigation was deemed not human participants research by the Tufts University Health Sciences institutional review board because it used publicly available data. Our study adhered to the Consolidated Health Economic Evaluation Reporting Standards (CHEERS) reporting guideline.

### Data Sources

Earnings from work (excluding income from other sources) among workers with ESI were obtained from the US Census Bureau’s Annual Social and Economic Supplements of the Current Population Survey (CPS).^16^ For consistency with premium costs for a family plan, we assessed family earnings rather than household or individual earnings. The US Census defines a family unit as “a group of 2 people or more related by birth, marriage, or adoption and residing together,”^17^ which is similar to the requirement most employers use for eligibility for a family plan. We used median wages to depict a “typical” family with ESI because mean wages can be skewed by the highest earners. Race and ethnicity, assessed as sociocultural constructs, were self-reported by CPS participants and evaluated for the head of household in each family.

Employer-sponsored health insurance premium costs from 1998 to 2019 were derived from the Kaiser Employer Health Benefits Survey.^5^ Since its inception in 1998, the annual Kaiser Employer Health Benefits Survey has reported trends in ESI. We extracted data on the national mean health care premium costs for an employer-sponsored family plan for each year from 1998 to 2019. To extend our analysis prior to 1998, we incorporated data from the Bureau of Labor Statistics’ Consumer Expenditure Survey (CES).^18^ The CES health care premium costs represent the mean premium costs from all sources, including ESI, Medicaid, and Medicare. Consequently, the mean health care premiums reported in the CES would underestimate ESI premiums. To account for this difference, we assessed overlapping years between the Kaiser and CES data from 1998 and 2010 (ending at 2010 due to passage of the Patient Protection and Affordable Care Act) to calculate the mean ratio between the Kaiser data and CES data premium costs. We then applied this ratio retrospectively to the years 1988 to 1998 in the CES data to estimate health care premium costs paid by the employee and employer during these years.

Health economists have demonstrated in multiple cases that employees bear the costs of both employee and employer contributions to ESI through reduced wages.^6,7,8,9,10,11^ Therefore, we included both employer-paid and employee-paid premiums because together they represent the full costs of a health care benefit package that could partially displace wages. Employer-sponsored health care insurance spending is not subject to income or payroll taxes. Other studies have shown that the displacement of wages associated with ESI costs is mitigated slightly by ESI’s tax deductibility^8,19^ and that reductions in wages may lag a few years because of employee contracts and employer uncertainty about health care cost changes. Thus, we assumed that health care premium increases would displace wages, although not exactly dollar-for-dollar owing to payroll taxation.^8^

### Statistical Analysis

Statistical analysis was conducted from February 2022 to July 2023. To estimate net premium costs, we accounted for the taxation on the additional wages earned if an employee hypothetically received the value of their health insurance as additional wages (and did not receive any health insurance). We subtracted this additional, hypothetical tax burden from the actual premium costs to calculate the net premium costs. We applied the federal payroll tax rate in each year from 1988 to 2019^20^ to the median annual earnings for our overall sample and to the median annual earnings for each subgroup (ie, Asian, Hispanic, non-Hispanic Black [hereafter, Black], and non-Hispanic White [hereafter, White] and the 20th, 40th, 60th, 80th, and 95th percentiles of earnings among families with ESI). The benefits of tax deductibility are greater for higher-paid workers who are taxed at a higher rate; thus, the net premium costs are higher for lower-paid workers after accounting for their tax deductibility. All analyses used the specific net health insurance premium costs for each subgroup.

We hereafter use the term *compensation* to describe the combined value of wages and health care premiums, while acknowledging that total compensation may include additional benefits, such as paid time off or retirement fund contributions. We recognized that wages and health care benefits are the major, but not only, components of a compensation package that employers offer. The annual dollar value of other employee benefits, including retirement fund contributions and paid time off, were not available for our study population for all years in the analysis. Other nationally representative analyses suggest that contributions to retirement funds have decreased during large portions of our study years, while paid time off as a percentage of employee compensation has remained relatively constant.^21,22^ We chose not to include out-of-pocket medical costs because these are distinct from employee compensation and because premiums are a fixed component of employee compensation. Given trends in increasing out-of-pocket medical spending,^23,24^ this exclusion of out-of-pocket costs likely underestimates the negative association of higher health care costs generally with family economic well-being.

To calculate the annual compensation package for the median US family with ESI, we summed median family earnings plus net premium costs for a family plan, adding contributions from both the employee and the employer. This approach recognizes that wages and health care premiums paid by an employer reasonably represent a compensation package that an employer is willing to pay, with employee premiums further offsetting wages for the employee.^8^

The primary outcome was the percentage of the employee compensation (ie, wages plus net premium costs) associated with health care premiums in each year among workers with ESI and then stratified by race and ethnicity (Asian, Black, Hispanic, and White) and by wage level (20th, 40th, 60th, 80th, and 95th percentiles of family earnings) among workers with ESI. In each year beginning in 1989, we also estimated potential lost annual earnings associated with increasing health care premiums based on the 1988 compensation. We first estimated counterfactual net premium costs in each year from 1989 to 2019 as if the percentage of employee compensation associated with health care premiums in each year remained at the 1988 levels. Then, we calculated the difference in the actual net premium costs and our counterfactual net premium costs based on the 1988 compensation package and report this difference as the lost earnings associated with increasing ESI costs in each year. Finally, we summed the lost earnings for all 32 years to report the total lost wages for the median family with ESI associated with increasing health insurance premiums over the study period. We used Wald tests to assess the statistical significance of the trends in each analysis, with a 2-sided *P* < .05 considered significant. Analyses were conducted in Stata SE, version 18 (StataCorp LLP) and Microsoft Excel (Microsoft Corp).

## Results

In 1988, 44.7 million people were covered by ESI family plans. The head of household had a mean (SD) age of 43.3 (13.1) years, 30.1% were female, and 2.4% identified as Asian, 8.6% as Black, 6.2% as Hispanic, and 82.8% as White (Table); the mean (SD) household size was 3.3 (1.3) people. In 2019, the population was generally similar, with 44.8 million people covered under ESI family plans. In 2019, the head of household had a mean (SD) age of 47.1 (12.9) years, 41.3% were female, and 1.3% identified as Asian, 9.9% as Black, 9.9% as Hispanic, and 78.9% as White; the mean (SD) household size was 3.4 (1.3) people. In 2019, the median US family with ESI earned approximately $17 000 more annually in 2019 US dollars than families in 1988 (median, $94 860 [IQR, $56 505-$151 168] vs $77 540 [IQR, $47 500-$112 400]).

**Table.** Sample Description of US Families Receiving Employer-Sponsored Health Insurance in 1988 and 2019^a^

Characteristic	US population covered by employer-sponsored health insurance family plans	
	1988 (N = 44 710 300)	2019 (N = 44 772 996)
Age, head of household, mean (SD), y	43.3 (13.1)	47.1 (12.9)
Female, head of household, %	30.1	41.3
Race and ethnicity, head of household, %		
Asian	2.4	1.3
Black	8.6	9.9
Hispanic	6.2	9.9
White	82.8	78.9
Household size, people, mean (SD)	3.3 (1.3)	3.4 (1.3)
Household earnings, median (IQR), 2019 US dollars	77 540 (47 500-112 400)	94 860 (56 505-151 168)

^a^ Sample means, medians, and percentages are calculated from the United States Census Bureau’s Current Population Survey. Survey weights are used to scale values to be nationally representative for the study sample.

In 1988, the median annual US family income among workers with ESI was $77 540 (IQR, $47 500-$112 400), and the mean health care premium cost paid by both employer and employee for a family plan was $6230 (95% CI, $6110-$6340) (all dollar amounts adjusted for inflation to 2019 dollars). Accounting for their tax deductibility, the net mean premium cost was $4485 (95% CI, $4400-$4570) in 1988. Starting in 1989 and for every year through 2019, health care premium growth was greater than earnings growth for US families with ESI. In 1988, health care premiums represented a mean of 7.9% (95% CI, 7.6%-8.2%) of compensation (wages plus premiums) and by 2019 increased significantly to a mean of 17.7% (95% CI, 17.0%-18.3%) of compensation (*P* < .001).

**Figure 1.**
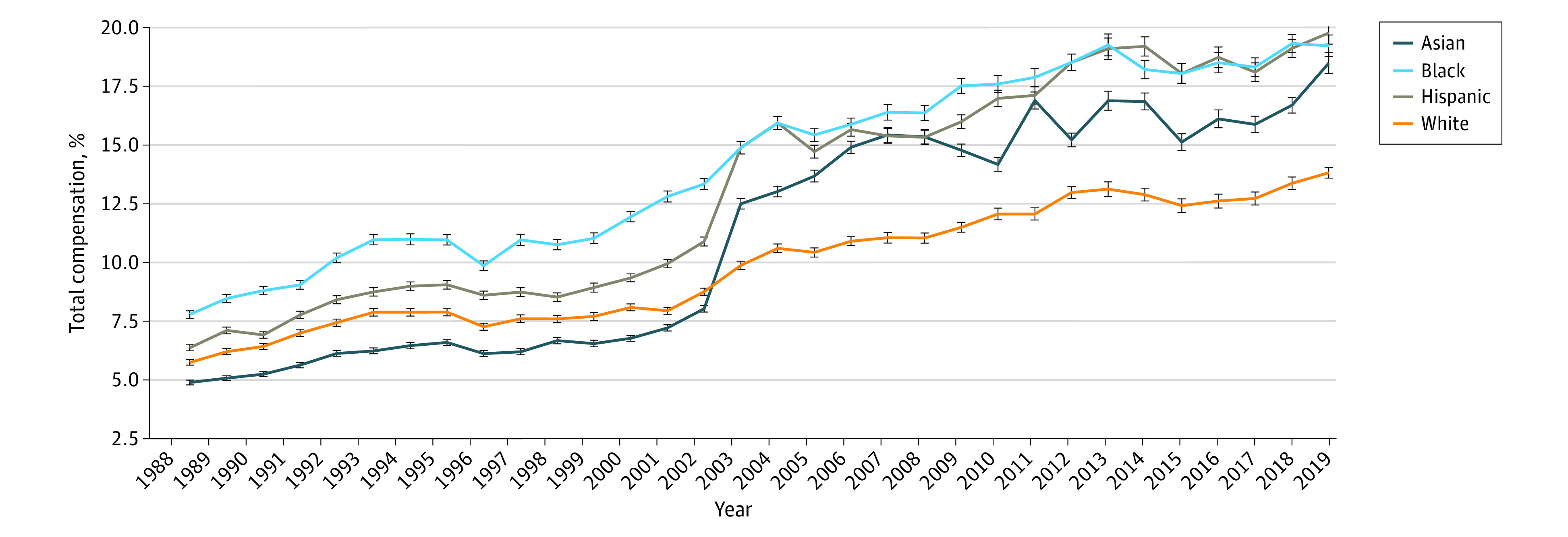
Percentage of Compensation Associated With Health Care Premiums Among Families With Employer-Sponsored Health Insurance From 1988 to 2019, by Race and Ethnicity Premium costs were derived from the Kaiser Employer Health Benefits Survey and Consumer Expenditure Survey. Net premium costs were calculated after accounting for federal payroll tax rates. Family earnings data by race and ethnicity were obtained from the US Census Bureau’s Annual Social and Economic Supplements of the Current Population Survey. Compensation refers to the sum of wages plus employer-sponsored premium costs, accounting for the pretax value of health care premiums. Error bars indicate 95% CIs. The difference between values in 1988 and 2019 for each group was statistically significant at *P* < .001. The US Census first assessed all 4 racial and ethnic categories included in our analysis in 1988, with changes introduced in 2003 that may affect how a respondent identifies.

In each year from 1988 to 2019, health care premiums represented a higher percentage of compensation for Black and Hispanic families than for Asian and White families among families with ESI (Figure 1). In 1988, health care premiums as a percentage of compensation were a mean of 7.8% (95% CI, 7.6%-8.0%) for Black families and 6.4% (95% CI, 6.2%-6.5%) for Hispanic families with ESI while proportionally less for Asian and White families at 4.9% (95% CI, 4.8%-5.0%) and 5.8% (95% CI, 5.6%-5.9%) of compensation, respectively (*P* < .001). Over 32 years, disparities increased in absolute 2019 dollars. By 2019, health care premiums as a percentage of compensation cost were a mean of 18.5% (95% CI, 18.0%-19.0%) for Asian families, 19.2% (95% CI, 18.8%-19.7%) for Black families, and 19.8% (95% CI, 19.3%-20.3%) for Hispanic families, while they were only 13.8% (95% CI, 13.5%-14.1%) for White families (*P* < .001).

**Figure 2.**
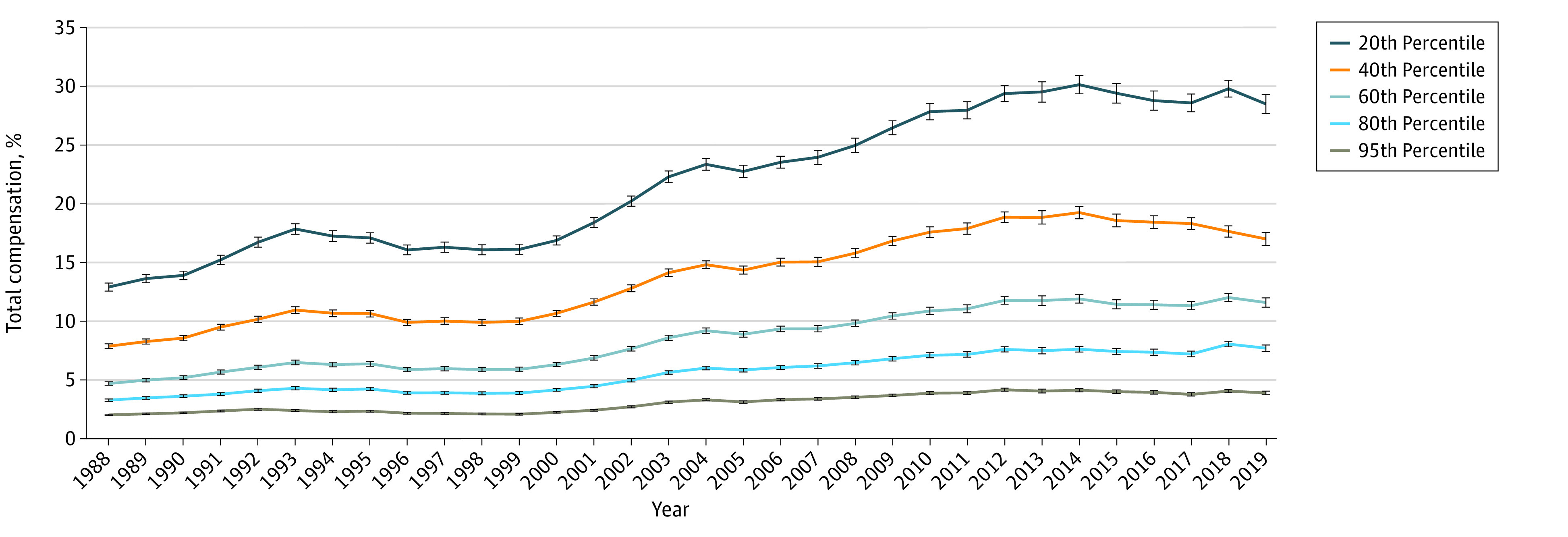
Percentage of Compensation Associated With Health Care Premiums Among Families With Employer-Sponsored Health Insurance From 1988 to 2019, by Earnings Percentile Premium costs were derived from the Kaiser Employer Health Benefits Survey and Consumer Expenditure Survey. Net premium costs were calculated after accounting for federal payroll tax rates. Family earnings data by earnings percentile were obtained from the US Census Bureau’s Annual Social and Economic Supplements of the Current Population Survey. Compensation refers to the sum of wages plus employer-sponsored premium costs, accounting for the pretax value of health care premiums. Error bars indicate 95% CIs. The difference between values in 1988 and 2019 for each group was statistically significant at *P* < .001.

Between 1988 and 2019, health care premiums more than doubled as a percentage of compensation for every earnings bracket among US families with ESI (Figure 2). However, in 2019, health care premiums as a percentage of compensation cost were only a mean of 3.9% (95% CI, 3.8%-4.0%) for families with ESI at the 95th percentile of earnings, an increase from 2.0% (95% CI, 1.9%-2.1%) in 1988. In contrast, health care premiums as a percentage of compensation for families with ESI at the 20th percentile of earnings were a mean of 28.5% in 2019 (95% CI, 27.8%-29.2%), an increase from 12.9% (95% CI, 12.7%-13.2%) in 1988. This inequity between earning brackets in health care premium expenditures as a percentage of compensation peaked in 2014, with modest decreases thereafter.

**Figure 3.**
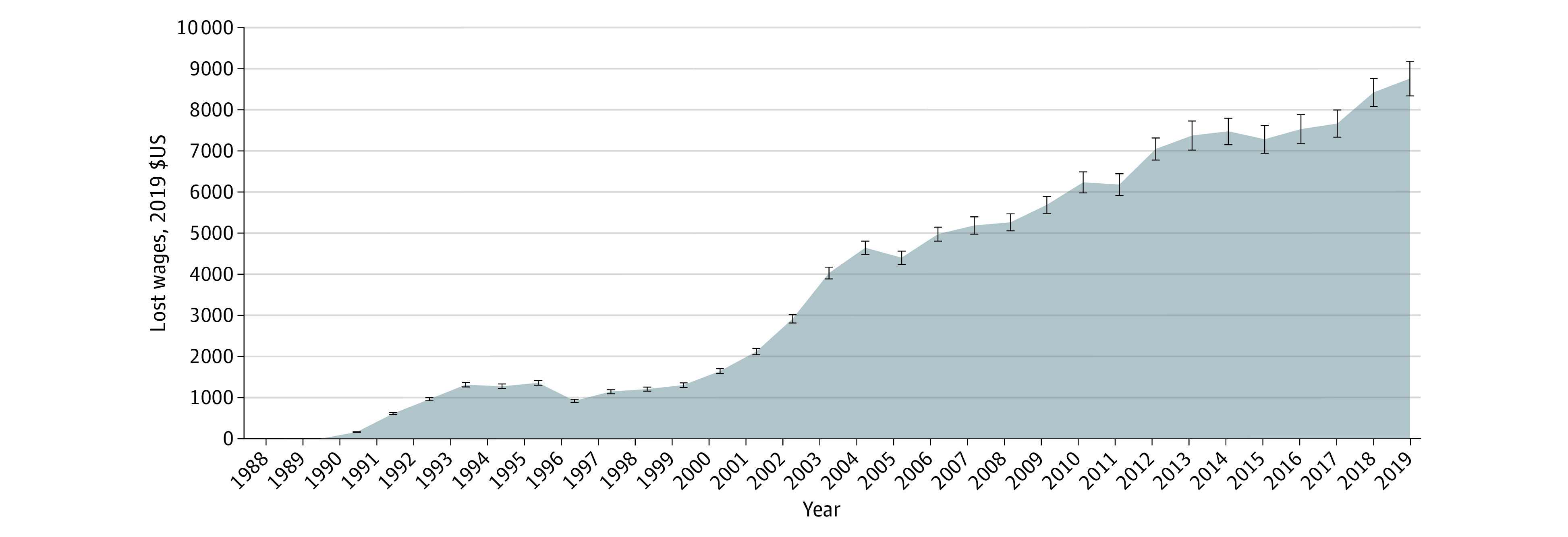
Lost Wages Associated With Health Care Premium Increases Among Families With Employer-Sponsored Health Insurance From 1988 to 2019 Premium costs were derived from the Kaiser Employer Health Benefits Survey and Consumer Expenditure Survey. Net premium costs were calculated after accounting for federal payroll tax rates. Family earnings data were obtained from the US Census Bureau’s Annual Social and Economic Supplements of the Current Population Survey. Based on the health care premium costs as a percentage of the compensation package in 1988 (5.5% of compensation), we calculated projected earnings for years 1989 to 2019 if the percentage of compensation associated with health care premiums remained constant. The lost wages associated with health care premium increases is the difference in projected vs observed earnings for years 1989 to 2019. The shaded area represents the cumulative lost wages, and the line represents the lost wages for each year; error bars indicate 95% CIs. The change from 1988 to 2019 was statistically significant at *P* < .001.

Over the course of these 32 years, the mean cumulative lost earnings associated with increasing health care premiums was $125 340 (95% CI, $120 155-$130 525), equal to 4.7% of total earnings for the median family receiving ESI during the study period (Figure 3). If health care premium costs for families with ESI had increased but remained at the same proportion of the 1988 compensation package (at 5.5%), then by 2019 the projected median US family earnings would have been $104 774 (95% CI, $102 262-$107 287), an $8774 (95% CI, $8354-$9195) increase over the observed median earnings of $96 000 (IQR, $56 000-$150 000). Disparities in lost wages by race and ethnicity were also evident, with the lost earnings associated with increasing premium costs representing 7.5% (95% CI, 7.3%-7.6%) of compensation for White families in 2019, whereas they represented 9.6% (95% CI, 9.4%-9.8%) of compensation for Black families and 9.3% (95% CI, 9.0%-9.5%) for Hispanic families with ESI.

To explore if household size may partially mitigate the inequities observed by race and ethnicity, we assessed household size among families with ESI. In 2019, White households receiving ESI had a mean (SD) household size of 3.2 (1.7) people, modestly smaller than for Asian (3.7 [1.8]), Black (3.4 [1.4]), or Hispanic (3.9 [1.5]) households.

## Discussion

Our analysis suggests that increasing health insurance premium costs were associated with decreased annual wages and increased earnings inequality by race and ethnicity and wage level and were likely associated with meaningful wage stagnation among US families receiving ESI. From 1988 to 2019, health insurance premiums increased from 7.9% of compensation (wages plus premiums) to 17.7% of compensation for US families with ESI. Moreover, Black and Hispanic families with ESI lost a higher percentage of their wages than White families with ESI to increasing health care premiums. By 2019, health care premiums as a percentage of compensation were 19.2% for Black families and 19.8% for Hispanic families, while they were only 13.8% for White families. We also observed large disparities in health care premiums by wage level. In 2019, health care premiums as a percentage of compensation represented 28.5% of compensation for families at the 20th percentile of earnings compared with only 3.9% for families at the 95th percentile of earnings—a 9-fold difference in the proportion of health care premiums as a percentage of compensation. The increasing health insurance premiums since 1988 contributed to nearly $9000 in annual lost earnings in 2019 and $125 000 in cumulative median lost earnings over the 32-year period. Several points require additional emphasis.

First, this study revealed that racial and ethnic earning disparities associated with health insurance premium growth have increased over time. Due to long-standing structural racism across the US economy, including in education and hiring, a larger proportion of Black and Hispanic workers than White workers have been employed in lower-paying jobs. This pattern holds true even among workers with ESI, as our analysis found. By receiving lower earnings historically, Black and Hispanic households shoulder a greater proportion of the increase in health care premiums as a percentage of their compensation, a trend that persisted throughout all 3 decades of our analysis. Thus, ESI may partially maintain interconnected historical inequities surrounding structural racism, earnings, and health care use. These findings are consistent with recent polling in which 60% of Black adults and 65% of Hispanic adults reported difficulty in affording health care costs compared with 39% of White adults.^1^

Second, the relative value of an ESI family plan depends partly on household size. Many plans have a set rate for families regardless of household size, meaning that larger households may receive a relatively better value on their premiums than smaller households who are paying similar costs. In 2019, White households receiving ESI had a mean (SD) household size of 3.2 (1.7) people, modestly smaller than for Asian (3.7 [1.8]), Black (3.4 [1.4]), or Hispanic (3.9 [1.5]) households. Thus, differences in the number of people covered could be considered to slightly mitigate the racial inequities of increasing premium costs, although the association with actual lost wages remains.

Third, the loss of $9000 in 2019 wages due to premium increases since 1989 has real consequences for US families, especially for those with economic insecurities. For example, one-third of US adults cannot afford a surprise $400 expense,^25^ and 1 in 5 US households has medical debt (median debt, $2000).^26^ As our analyses found, increasing premium costs are particularly problematic for lower-paid workers, which could place their families at greater risk of economic instability.

Fourth, this study builds on existing literature showing that increasing premium costs are associated with wage stagnation.^6,7,8,9,10,11^ For example, previous studies found that a 10% increase in health insurance premiums reduced wages by 2.3%^9^ and that about two-thirds of health insurance premium growth was compensated through lower wages^10^ (consistent with our analysis when accounting for tax deductibility of health care premiums). Another analysis concluded that increasing health care costs from 1999 to 2009 caused families to lose an estimated $5600 per year in wages by 2009.^8^ Faced with excessive premium growth, some employers are also responding by pivoting to greater employee cost sharing through high-deductible plans, reducing plan generosity, or by shifting employees to part-time work.^6,9,27^ Our finding that families with ESI may have lost up to $125 000 over 3 decades due to increasing premium costs is consistent with former studies^6,7,8,9,10^ and provides important new evidence on long-term associations over a 30-year career.

Fifth, few ESI plans vary premium costs by the wage level of the employee,^12^ making increasing health care premiums more burdensome for lower-wage workers. Our findings should be interpreted to apply to most ESI plans that do not vary premium costs by wage level. Plans that offer more affordable premiums to lower-wage workers could help improve earnings inequality amid increasing health care premiums. However, even among the few large employers that adjust premium costs by earnings level, the relative price of premiums is often not directly proportional to the earnings gap between the highest-paid and lowest-paid workers in the company,^28^ meaning low-income workers still pay a greater percentage of their income to premiums.

Our findings that lower-wage workers contribute more to ESI premiums as a percentage of their compensation aligns with evidence that, across the entire US health care system, the burden of health care financing is greater for lower-income than higher-income households.^29^ Economists, such as Kaestner and Lubotsky,^30^ have further noted that the tax deductibility of health care premiums has a modest adverse association with income inequality because the net premium costs (ie, pretax value) are effectively lower for employees in the highest tax brackets. They found that eliminating the tax deductibility of health care premiums reduces the ratio of the 90th to the 10th percentile after-tax income distribution by approximately 4%.^30^ Others have commented that ESI taxation in practice could be regressive because health benefits account for a larger share of compensation for low-income employees who thus would pay a higher percentage of their income to taxes.^31^

Other nations, such as Canada and England, have a health insurance system that is separate from employment. Expanding US public insurance programs, such as Medicaid and Medicare, has been promoted by some as a means to improve equity.^32^ However, even with increasing premium costs, recent surveys show high worker satisfaction with ESI coverage.^33^ Prior to the passage of the Patient Protection and Affordable Care Act, ESI was voluntarily provided to recruit and retain talented employees. The Patient Protection and Affordable Care Act now requires companies with 50 or more employees to provide basic health insurance coverage,^34^ although companies still compete on the overall benefits and costs of such coverage.

### Strengths and Limitations

Our investigation has several strengths. First, our study adds important new evidence that increasing premium costs may increase earnings inequality, including by race and ethnicity, among families receiving ESI. Second, to our knowledge, this is the first study to assess 30-year growth in the percentage of compensation associated with ESI premium costs and its association with wage stagnation among US families with ESI. Third, we combined data from large, nationally representative data sets on ESI costs and family earnings to focus on families with ESI and potential disparities. Fourth, the analysis incorporated the full costs of ESI premiums by including costs paid by employees and their employers. Fifth, our analyses accounted for relevant payroll taxation rates in each year for each subgroup’s annual earnings. This approach acknowledges that employers do not pay taxes on premiums, and thus net premium costs are lower than would appear without consideration of taxation.

Our analysis also has some limitations. First, our study design cannot determine causality, and employers may not redirect all savings associated with slower premium growth toward increased employee pay. For example, employers may instead invest in capital, new business ventures, or an expanded workforce. Nonetheless, premium growth represents an opportunity loss for higher wages within the budgeted costs for employee compensation, and prior research has documented associations between health care premium increases and depressed wages.^6,7,8,9,10,11^ Second, increasing premiums could be offset by reductions in other employer-sponsored benefits, such as vacation time or retirement fund contributions; however, data limitations prevented us from exploring these associations. Historical trends support potential decreasing employer contributions to retirement funds and unchanged amounts of paid time off as a percentage of employee compensation over large portions of the study period.^21,22^ Thus, our findings may underestimate the true economic costs to families of increasing ESI premiums if greater premium costs not only cut into wages but also degrade the generosity of other benefits because employers have a finite amount of money to contribute to combined employee pay and benefits. Whether premium growth has also exacerbated racial and ethnic wealth gaps in retirement savings is an area for future research. Third, these findings are applicable to US families with ESI family plans with fixed premium costs for all earnings levels and are not generalizable to US families with non-ESI plans nor to uninsured families. Fourth, some employees may be willing to receive lower wages if costlier premiums buy higher-quality health care, such as coverage of new medications and treatments. Although health care spending increased faster in the US than in other high-income countries during our 32-year study period, the US continues to have the highest maternal and infant mortality, the lowest life expectancy at birth, and the highest death rates for avoidable or treatable conditions among high-income countries, suggesting that increased health care spending does not guarantee better population-level health outcomes.^35^ Fifth, we did not incorporate state income tax rates in our net premium cost calculations. However, state income taxes are typically small compared with federal payroll taxes (the mean state income tax in 2022 was only 2.1%),^36^ and a handful of states do not levy income taxes; thus, their inclusion would likely have little effect on our findings. Sixth, our analysis does not account for increasing personal deductibles and other out-of-pocket medical expenses; thus, our findings likely underestimate the full negative association of increasing health care costs with economic disparities by race and ethnicity and wage level.

## Conclusions

This economic evaluation of US families with ESI suggests that increasing health care premiums were associated with decreased annual earnings and increased earnings inequality, including by race and ethnicity and wage level, and were also associated with reduced median cumulative family earnings by approximately $125 000 over 32 years. Our results depict the hidden costs of increasing health insurance premiums for the US worker: less opportunity for wage growth and a heavier burden of health insurance premiums on lower-paid workers and on Black and Hispanic workers. Our analysis suggests a need for future research and for a corresponding US health care policy to examine the role that increasing health care premiums play in stagnating employee wages and increasing income inequality.

## Notes

Supplement.
Data Sharing Statement
