# Supplementary material for: Employer-Sponsored Health Insurance Premium Cost Growth and Its Association With Earnings Inequality Among US Families
Source: JAMA Netw Open. 2024 Jan 16;7(1):e2351644. doi: 10.1001/jamanetworkopen.2023.51644 (PMC10792464; doi:10.1001/jamanetworkopen.2023.51644)
Supplement: Supplement. — Data Sharing Statement [file jamanetwopen-e2351644-s001.pdf]

Data Sharing Statement

Hager. Employer-Sponsored Health Insurance Premium Cost Growth and Its Association With Earnings Inequality Among US Families. *JAMA Netw Open*. Published January 16, 2024. doi:10.1001/jamanetworkopen.2023.51644

Data
Data available: Yes
Data types: Data (not involving human participants)
How to access data: The data used for this study are publicly available from the following sources: Annual Social and Economic Supplements. United States Census Bureau. Accessed 7/20/2023, https://www.census.gov/data/datasets/time-series/demo/cps/cps-asec.html Employer Benefits Survey, 2019. Henry J. Kaiser Family Foundation. Accessed 7/20/2023, https://www.kff.org/health-costs/report/2019-employer-health-benefits-survey/ Consumer Expenditure Survey Multiyear Tables. United States Bureau of Labor Statistics. Accessed 7/20/2023, https://www.bls.gov/cex/tables.htm. Historical U.S. Federal Individual Income Tax Rates & Brackets, 1862-2021. Tax Foundation. Accessed 7/20/2023, https://taxfoundation.org/historical-income-tax-rates-brackets/
When available: With publication

Supporting Documents
Document types: None

Additional Information
Who can access the data: The data used for this study are already publicly available from the United States Census Bureau, United States Bureau of Labor Statistics, the Kaiser Family Foundation, and the Tax Foundation.
Types of analyses: The data used for this study are already publicly available from the United States Census Bureau, United States Bureau of Labor Statistics, the Kaiser Family Foundation, and the Tax Foundation.
Mechanisms of data availability: The data used for this study are already publicly available from the United States Census Bureau, United States Bureau of Labor Statistics, the Kaiser Family Foundation, and the Tax Foundation.
